# Poor glycemic control is independently associated with fibrinolysis shutdown in patients with peripheral artery disease

**DOI:** 10.1016/j.jvssci.2026.100441

**Published:** 2026-08-24

**Authors:** Swechha Bhatt, Aseman Bagheri Sheshdeh, Shezan Fouzdar, Radha Bansal, Prajakta Lokhande, Shanaya Vimadalal, Shiv Patel, Anahita Dua

**Affiliations:** Division of Vascular Surgery, Department of Surgery, Massachusetts General Hospital/Harvard Medical School, Boston, MA

**Keywords:** Fibrinolysis, Glycated hemoglobin, Hyperglycemia, Peripheral arterial disease, Plasminogen activator inhibitor-1, Thromboelastography

## Abstract

**Objective:**

Fibrinolysis plays a critical role in regulating thrombotic potential by facilitating the breakdown of fibrin-rich thrombi after clot formation. Impaired fibrinolysis, often referred to as hypofibrinolysis or fibrinolytic shutdown, results in reduced clot degradation and promotes thrombus persistence, propagation, and recurrent vascular occlusion. Consequently, impaired fibrinolysis is increasingly recognized as an important contributor to thrombotic risk in patients with peripheral artery disease (PAD). Poor glycemic control has been associated with a prothrombotic milieu through mechanisms including enhanced platelet activation, increased fibrin clot density, and reduced fibrinolytic capacity; however, its relationship with fibrinolytic shutdown in PAD remains poorly defined. Therefore, we aimed to evaluate the association between glycemic control and fibrinolytic shutdown using viscoelastic testing.

**Methods:**

We performed a retrospective cohort study of 192 patients with PAD who underwent revascularization. Patients were stratified by glycated hemoglobin (HbA1c) < 7% and ≥7%. Viscoelastic testing was performed using thromboelastography. Fibrinolysis was assessed using lysis at 30 minutes, and phenotypes were defined as severe shutdown (≤0.3%), partial shutdown (0.3%-0.8%), physiologic (0.8%-3.0%), and hyperfibrinolysis (>3.0%). Multivariable logistic regression evaluated the association between HbA1c and severe fibrinolysis shutdown, adjusting for demographic, clinical, and treatment variables.

**Results:**

Patients with HbA1c ≥ 7% demonstrated lower lysis at 30 minutes values compared with those with HbA1c < 7% (median, 0.00 [interquartile range, 0.00-0.35] vs median, 0.30 [interquartile range, 0.00-0.90]; *P* = .015) and a higher prevalence of severe fibrinolysis shutdown (74.7% vs 51.3%; *P* = .009). After adjustment, patients with HbA1c ≥ 7% had a higher predicted probability of severe shutdown (68.8% [95% CI, 51.4-82.2] vs 41.0% [CI, 26.3-57.4]; *P* < .001). In continuous analysis, each 1% increase in HbA1c was associated with increased odds of severe shutdown (adjusted odds ratio, 1.50; 95% CI, 1.19-1.93; *P* = .001). Severe shutdown was additionally associated with thrombosis-driven reintervention (22.2% vs 8.0%; adjusted odds ratio, 3.60; 95% CI, 1.42-10.54; *P* = .011).

**Conclusions:**

Poor glycemic control was independently associated with severe fibrinolysis shutdown in patients with PAD, and severe shutdown was associated with increased thrombosis-driven reintervention. A dose-dependent relationship between HbA1c and impaired fibrinolysis suggests a mechanistic link between hyperglycemia and prothrombotic risk. Hence, patients with higher glucose levels may need increased thromboprophylaxis strategies to mitigate thrombotic risk. These findings may have implications for risk stratification and targeted therapeutic strategies.

***Clinical relevance*:**

Fibrinolysis shutdown is an underrecognized contributor to residual thrombotic risk in peripheral artery disease (PAD), yet it is not captured by conventional risk assessment tools. This study demonstrates that poor glycemic control (glycated hemoglobin ≥ 7%) is independently and dose-dependently associated with severe fibrinolysis shutdown on thromboelastography in patients with PAD. These findings suggest that hyperglycemia contributes to a prothrombotic hemostatic phenotype distinct from platelet-mediated pathways targeted by standard antiplatelet therapy. Viscoelastic testing may therefore identify a subset of patients with PAD, particularly those with suboptimal glycemic control who could benefit from closer surveillance, metabolic optimization, or individualized antithrombotic strategies to reduce thrombotic complications and adverse limb events.

**Article Highlights**

• **Type of Research:** Single-center retrospective cohort study evaluating the association between glycemic control and fibrinolysis shutdown using viscoelastic testing
• **Key Findings:** Analysis of 192 patients with peripheral artery disease (PAD) stratified by glycated hemoglobin (HbA1c <7% vs ≥ 7%) demonstrated that patients with HbA1c ≥ 7% had significantly lower lysis at 30 minutes values (median, 0.00 [interquartile range, 0.00-0.35] vs median, 0.30 [interquartile range, 0.00-0.90]; *P* = .015) and a higher prevalence of severe fibrinolysis shutdown (74.7% vs 51.3%; *P* = .009) on thromboelastography. After multivariable adjustment, HbA1c ≥ 7% remained independently associated with severe fibrinolysis shutdown (adjusted odds ratio, 3.18; 95% CI, 1.64-6.43; *P* < .001), and each 1% increase in HbA1c was associated with 50% higher odds of severe shutdown (adjusted odds ratio, 1.50; 95% CI, 1.19-1.93; *P* = .001).
• **Take Home Message:** Poor glycemic control is independently and dose-dependently associated with fibrinolysis shutdown in patients with peripheral artery disease. Severe fibrinolysis shutdown was also associated with increased thrombosis-driven reintervention, supporting further investigation of fibrinolytic phenotype as a potential marker of thrombotic risk.

Peripheral artery disease (PAD) affects an estimated 230 million individuals worldwide and is associated with a substantially elevated risk of limb-threatening ischemia, major adverse cardiovascular events (MACEs), and death.^1^^,^^2^ Despite advances in antithrombotic therapy and revascularization techniques, residual thrombotic risk remains high in this population, and adverse limb events continue to occur even among patients receiving guideline-directed medical therapy.^3^ This suggests that PAD-associated thrombosis is driven by pathophysiological mechanisms not fully captured by conventional risk stratification or adequately addressed by standard antiplatelet and anticoagulant strategies.

Fibrinolysis, the enzymatic process by which plasmin degrades fibrin clots plays a central and underappreciated role in maintaining vascular patency. Dysregulation of fibrinolysis, particularly impaired clot dissolution, may contribute directly to thrombotic complications in patients with arterial disease.^4^ Viscoelastic testing, including thromboelastography (TEG), offers a functional, point-of-care assessment of the entire hemostatic process, including fibrinolysis, through parameters such as lysis at 30 minutes (LY30), the percentage of clot LY30 after maximum clot strength.^5^ Fibrinolysis shutdown, defined by LY30 ≤ 0.8% and most severely at ≤0.3%, has emerged as a clinically distinct phenotype initially characterized in trauma patients and associated with prothrombotic complications including venous thromboembolism and impaired clot resolution.^6^^,^^7^ However, the relevance of this phenotype in patients with chronic arterial disease remains largely unexplored.

Hyperglycemia is a potent modulator of hemostasis. Type 2 diabetes and chronic hyperglycemia are associated with increased expression of plasminogen activator inhibitor-1 (PAI-1), impaired tissue-type plasminogen activator (tPA) activity, altered fibrin network architecture favoring denser and more fibrinolysis-resistant clots, and enhanced platelet reactivity.^8^, ^9^, ^10^, ^11^ Together, these mechanisms create a hypofibrinolytic, prothrombotic milieu that may be particularly detrimental in the context of underlying arterial disease. Despite the biological plausibility of a link between glycemic dysregulation and impaired fibrinolysis, this relationship has not been systematically characterized in patients with PAD using functional hemostatic testing.

Glycated hemoglobin (HbA1c) provides an integrated measure of glycemic exposure over the preceding 2 to 3 months and serves as the clinical standard for assessing long-term glycemic control.^12^ We hypothesized that HbA1c, as a proxy for chronic hyperglycemia, would be independently associated with fibrinolysis shutdown in patients with PAD. Accordingly, the objective of this study was to evaluate the association between glycemic control, stratified by HbA1c, and fibrinolysis phenotype assessed by viscoelastic testing in a retrospective cohort of patients with established PAD.

## Methods

### Study design and population

We conducted a retrospective cohort study of patients with PAD undergoing clinical evaluation with viscoelastic testing at a single academic vascular center. A total of 192 consecutive patients who met inclusion criteria were identified from an institutional database and included in the analysis. Patients with PAD confirmed by previous imaging, ankle-brachial index measurement, or previous vascular intervention were eligible. There were no restrictions based on PAD severity, previous intervention, or concurrent cardiovascular diagnoses. Patients were included when an HbA1c measurement was available within 90 days of TEG testing, allowing glycemic control to be evaluated in relation to the contemporaneous fibrinolytic phenotype. Simultaneous serum glucose measurements were not uniformly available at the time of TEG testing and were therefore not included in the primary analysis. Glycemic control was categorized according to American Diabetes Association criteria, with HbA1c < 7% defined as controlled glycemia and HbA1c ≥ 7% as suboptimal glycemic control.

This study was approved by the Mass General Brigham Institutional Review Board (IRB Nos. 2022P002264, 2022P001918, and 2023P002724). Informed written or electronic informed consent was obtained from all patients or their legally authorized representatives. Clinical and viscoelastic data were maintained in an institutional study database.

### Data collection

Baseline demographic and clinical characteristics were extracted from the electronic medical record, including age, sex, race and ethnicity, body mass index (BMI), smoking status, cardiovascular comorbidities (such as hypertension, hyperlipidemia, coronary artery disease, previous myocardial infarction, and previous stroke), and thromboembolic history (ie, deep vein thrombosis and pulmonary embolism). Sex was ascertained from administrative data in the electronic medical record. Vascular disease burden was categorized as PAD alone, PAD with coronary artery disease or stroke, or PAD with both coronary artery disease and stroke. Renal function was categorized using estimated glomerular filtration rate: ≥90, 60-89, 30-59, 15-29, or <15 mL/min/1.73 m^2^ (including dialysis). Medication exposure at the time of viscoelastic testing, including antiplatelet therapy (aspirin, clopidogrel, and ticagrelor) and anticoagulant use, was recorded.

For each patient, the HbA1c measurement closest to TEG testing and obtained within 90 days was used for analysis. This interval was selected because HbA1c reflects integrated glycemic exposure over the preceding approximately 2 to 3 months, thereby providing a measure of chronic glycemic burden relevant to the time of TEG assessment.

### Viscoelastic testing and fibrinolysis phenotype definitions

Fibrinolysis was assessed using TEG (Haemonetics Corporation). The primary parameter of interest was LY30, defined as the percentage of clot LY30 after maximum clot strength. Fibrinolysis phenotypes were defined based on predefined LY30 thresholds as follows: severe shutdown (LY30 ≤ 0.3%), partial shutdown (LY30, 0.3-0.8%), physiologic fibrinolysis (LY30, 0.8–3.0%), and hyperfibrinolysis (LY30 > 3.0%). Severe fibrinolysis shutdown was selected as the primary outcome of interest based on its established association with prothrombotic complications in previous literature.^6^^,^^7^ Additional TEG parameters collected included reaction time, functional fibrinogen level, and citrated functional fibrinogen maximum amplitude.

### Statistical analysis

Continuous variables were assessed for normality by visual inspection of histograms and quantile-quantile plots and are reported as medians with interquartile ranges (IQRs). Categorical variables are presented as counts and percentages. Comparisons between glycemic control groups were performed using the Wilcoxon rank-sum test for continuous variables and the *χ*^2^ test or Fisher exact test for categorical variables, as appropriate.

The association between glycemic control and fibrinolysis was evaluated using both continuous and categorical approaches. LY30 values were compared directly between groups, and fibrinolysis phenotypes were analyzed as categorical outcomes. Multivariable logistic regression models were used to evaluate the association between HbA1c and severe fibrinolysis shutdown, with HbA1c modeled both as a continuous variable (per 1% increase) and as a binary variable (HbA1c ≥ 7% vs <7%). Models were adjusted a priori for clinically relevant covariates, including age, sex, BMI, smoking status, renal function, vascular disease burden, antiplatelet therapy, and anticoagulant use. To account for potential confounding by diabetes treatment intensity, a sensitivity analysis additionally incorporated glucose-lowering medication burden and insulin therapy. Medication burden was categorized as no therapy, single-agent therapy, or multiple-agent therapy (≥2 agents) based on use of insulin, biguanides, sodium-glucose cotransporter-2 inhibitors, glucagon-like peptide-1 receptor agonists, and dipeptidyl peptidase-4 inhibitors. Insulin therapy was additionally evaluated as an independent covariate given its potential association with diabetes severity.

Adjusted predicted probabilities of severe fibrinolysis shutdown were estimated using marginal means derived from the fitted multivariable model. Odds ratios (ORs) with 95% confidence intervals (CIs) were reported. Clinical outcomes following the index vascular intervention were additionally assessed, including thrombosis-driven reintervention, major amputation, and MACEs. Thrombosis-driven reintervention was defined as repeat surgical or endovascular intervention required for a clinically significant thrombotic event following the index peripheral vascular intervention.

All statistical tests were two-sided, and a *P* value of less than .05 was considered statistically significant. Analyses were performed using R statistical software (version 4.5.2; R Foundation for Statistical Computing).

## Results

### Patient characteristics

A total of 192 patients were included in the analysis, of whom 113 (58.9%) had HbA1c < 7% and 79 (41.1%) had HbA1c ≥ 7%. The median age of the overall cohort was 70.0 years (IQR, 63.0-75.5), with no significant difference between groups (71.0 vs 68.0 years; *P* = .078). The cohort had similar sex distribution across glycemic groups (*P* = .90). Baseline characteristics are presented in Table I.

**Table I:** Baseline demographic, clinical, and viscoelastic characteristics stratified by glycemic control in patients with peripheral artery disease (*PAD*)

Variables	Overall (N = 192)	HbA1c < 7% (N = 113)	HbA1c ≥ 7% (N = 79)	*P* value
Age, years	70.0 [63.0-75.5]	71.0 [64.0-77.0]	68.0 [63.0-74.0]	.078
Sex				.90
Female	132 (69)	77 (68)	55 (70)	
Male	60 (31)	36 (32)	24 (30)	
BMI, kg/m^2^	27.15 [24.02-30.35]	26.22 [23.24-29.99]	28.40 [24.86-31.15]	.034
Hypertension	181 (94)	106 (94)	75 (95)	>.90
Hyperlipidemia	174 (91)	101 (89)	73 (92)	.60
Tobacco use				.30
Current smoker	35 (18)	21 (19)	14 (18)	
Former smoker	117 (61)	73 (65)	44 (56)	
Nonsmoker	40 (21)	19 (17)	21 (27)	
Renal status (eGFR, mL/min/1.73 m^2^)				.50
≥90	79 (41)	51 (45)	28 (35)	
60-89	51 (27)	26 (23)	25 (32)	
30-59	40 (21)	23 (20)	17 (22)	
15-29	7 (3.6)	3 (2.7)	4 (5.1)	
<15 or dialysis	15 (7.8)	10 (8.8)	5 (6.3)	
Coronary artery disease	130 (68)	77 (68)	53 (67)	.90
History of MI	69 (36)	37 (33)	32 (41)	.30
History of stroke	39 (20)	23 (20)	16 (20)	>.90
DVT	36 (19)	19 (17)	17 (22)	.50
PE	4 (2.1)	1 (0.9)	3 (3.8)	.30
Vascular disease burden				.60
PAD only	47 (24)	29 (26)	18 (23)	
PAD + CAD/stroke	121 (63)	68 (60)	53 (67)	
PAD + CAD + stroke	24 (13)	16 (14)	8 (10)	
Glucose-lowering therapy				
Insulin therapy	95 (49.5)	34 (30.1)	61 (77.2)	**<.001**
Biguanide	50 (26.0)	21 (18.6)	29 (36.7)	**.007**
SGLT2 inhibitor	13 (6.8)	6 (5.3)	7 (8.9)	.388
GLP-1 receptor agonist	24 (12.5)	7 (6.2)	17 (21.5)	**.003**
DPP-4 inhibitor	17 (8.9)	5 (4.4)	12 (15.2)	**.018**
Glucose-lowering medication burden				**<.001**
None	58 (30.2)	54 (47.8)	4 (5.1)	
Single agent	80 (41.7)	45 (39.8)	35 (44.3)	
Multiple agents (≥2)	54 (28.1)	14 (12.4)	40 (50.6)	
Aspirin	149 (78)	83 (73)	66 (84)	.11
Clopidogrel	73 (38)	43 (38)	30 (38)	>.90
Anticoagulant therapy	71 (37)	41 (36)	30 (38)	.90
Antiplatelet regimen				.60
Single antiplatelet	115 (60)	67 (59)	48 (61)	
Dual antiplatelet	55 (29)	31 (27)	24 (30)	
None	22 (11)	15 (13)	7 (8.9)	
Reaction time, min	6.40 [5.10-7.70]	6.60 [5.20-7.60]	6.30 [5.10-7.70]	.60
CFF MA, mm	23.95 [18.95-33.05]	23.40 [18.40-30.50]	24.90 [19.30-34.30]	.14
FLEV, mg/dL	431.55 [345.80-603.10]	421.50 [335.80-556.60]	454.40 [352.20-625.90]	.11

*BMI*, Body mass index; *CAD*, coronary artery disease; *CFF MA*, citrated functional fibrinogen maximum amplitude; *DPP-4*, dipeptidyl peptidase-4; *DVT*, deep vein thrombosis; *eGFR*, estimated glomerular filtration rate; *FLEV*, functional fibrinogen level; *GLP-1*, glucagon-like peptide-1; *HbA1c*, glycated hemoglobin; *MI*, myocardial infarction; *PAD*, peripheral artery disease; *PE*, pulmonary embolism; *SGLT2*, sodium-glucose cotransporter-2.

Values are presented as median [interquartile range] for continuous variables and n (%) for categorical variables. Comparisons between groups used the Wilcoxon rank-sum test for continuous variables and *χ*^2^ or Fisher exact test for categorical variables, as appropriate. Glucose-lowering medication burden was categorized as no therapy, single-agent therapy, or multiple-agent therapy (≥2 agents) based on the use of insulin, biguanides, SGLT2 inhibitors, GLP-1 receptor agonists, and DPP-4 inhibitors. HbA1c <7% denotes controlled glycemia; HbA1c ≥ 7% denotes suboptimal glycemic control.

Boldface *P* values indicate statistical significance.

Patients with HbA1c ≥ 7% had a significantly higher BMI compared with those with HbA1c < 7% (BMI, 28.4 vs 26.2 kg/m^2^; *P* = .034). There were no significant differences between groups in race/ethnicity, prevalence of hypertension (94% vs 95%), hyperlipidemia (89% vs 92%), coronary artery disease (68% vs 67%), previous myocardial infarction (33% vs 41%), previous stroke (20% vs 20%), deep vein thrombosis (17% vs 22%), or pulmonary embolism (0.9% vs 3.8%; all *P* > .05). Smoking status, renal function, and vascular disease burden were also comparable between groups, with the majority of patients in both groups exhibiting polyvascular disease.

Medication use was balanced between glycemic groups, including aspirin use (73% vs 84%; *P* = .11), clopidogrel (38% vs 38%; *P* > .90), anticoagulation therapy (36% vs 38%; *P* = .90), and antiplatelet regimen distribution (*P* = .60). Baseline viscoelastic parameters were comparable between groups, with no significant differences in reaction time (6.6 vs 6.3 minutes; *P* = .60), citrated functional fibrinogen maximum amplitude (23.4 vs 24.9 mm; *P* = .14), or functional fibrinogen levels (421.5 vs 454.4 mg/dL; *P* = .11).

### Fibrinolysis parameters by glycemic control

Patients with HbA1c ≥ 7% demonstrated significantly lower LY30 values compared with those with HbA1c < 7% (median, 0.00 [IQR, 0.00-0.35] vs median, 0.30 [IQR, 0.00-0.90]; *P* = .015), indicating reduced fibrinolytic activity (Table II). Fibrinolysis phenotype distribution differed significantly between groups (*P* = .009). Patients with HbA1c ≥ 7% had a higher prevalence of severe fibrinolysis shutdown (74.7% vs 51.3%), whereas physiologic fibrinolysis (8.9% vs 22.1%) and partial shutdown (13.9% vs 22.1%) were less common in the suboptimal glycemic control group. Hyperfibrinolysis was infrequent in both groups (2.5% vs 4.4%). The distribution of fibrinolysis phenotypes is illustrated in Fig 1.

**Table II:** Association of glycemic control with fibrinolysis parameters and phenotype assessed by viscoelastic testing

Variables	HbA1c < 7%	HbA1c ≥ 7%	*P* value
LY30, %	0.30 [0.00-0.90]	0.00 [0.00-0.35]	.015
Fibrinolysis phenotype			.009
Severe shutdown (≤0.3%)	58 (51.3)	59 (74.7)	
Partial shutdown (0.3%-0.8%)	25 (22.1)	11 (13.9)	
Physiologic (0.8%-3.0%)	25 (22.1)	7 (8.9)	
Hyperfibrinolysis (>3.0%)	5 (4.4)	2 (2.5)	

*LY30*, Lysis at 30 minutes; *HbA1c*, glycated hemoglobin.

Fibrinolysis phenotypes were defined based on LY30 thresholds: severe shutdown (LY30 ≤ 0.3%), partial shutdown (0.3%-0.8%), physiologic fibrinolysis (0.8%-3.0%), and hyperfibrinolysis (>3.0%). Continuous LY30 values compared using the Wilcoxon rank-sum test; fibrinolysis phenotype distribution compared using Pearson *χ*^2^ test.

Categorical data are shown as number (%).

**Fig 1:**
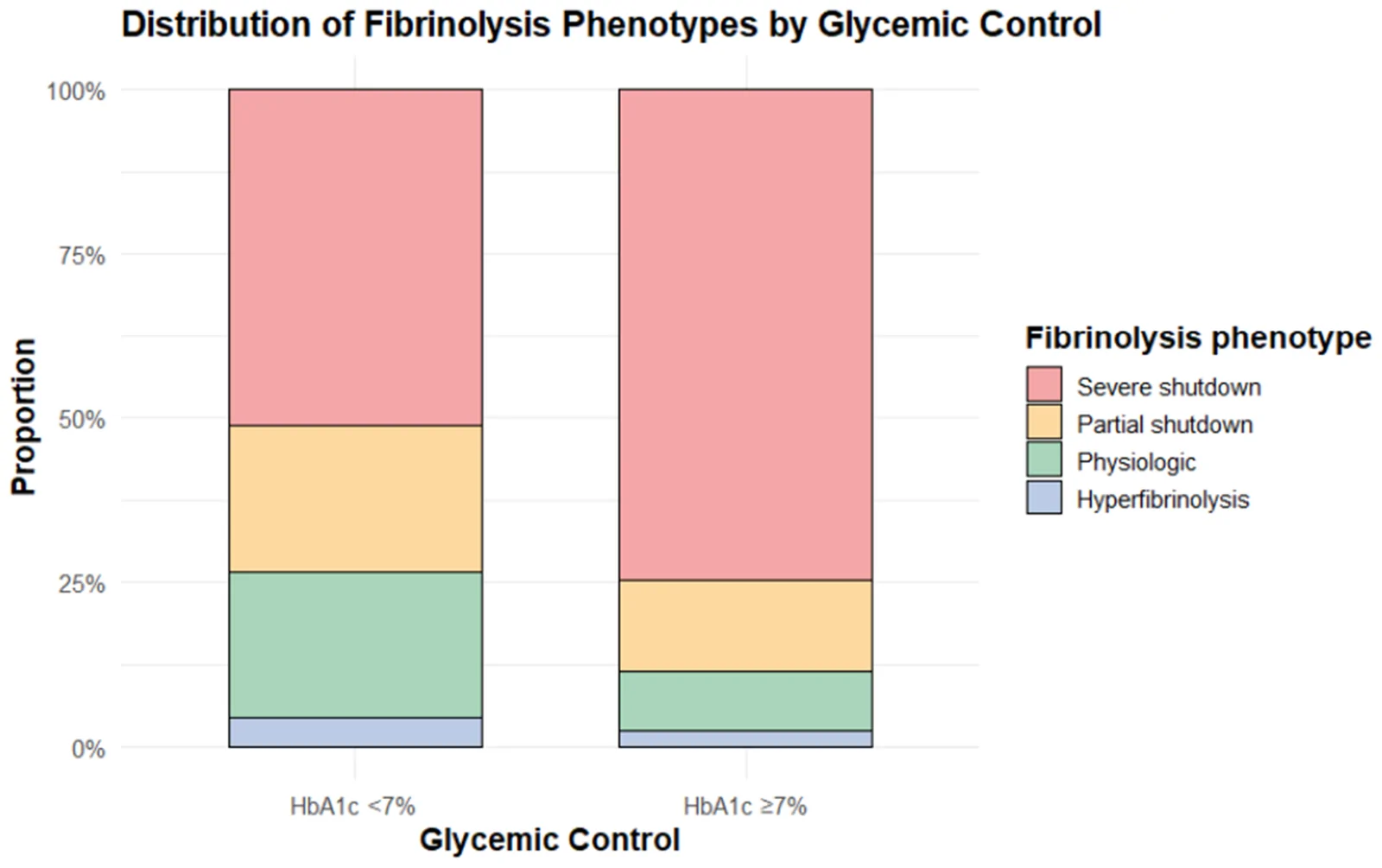
Distribution of fibrinolysis phenotypes according to glycemic control. Stacked bar chart illustrating the distribution of fibrinolysis phenotypes stratified by glycemic control. Fibrinolysis phenotypes were defined according to lysis at 30 minutes (LY30) as severe fibrinolysis shutdown (≤0.3%), partial shutdown (0.3%-0.8%), physiologic fibrinolysis (0.8%-3.0%), and hyperfibrinolysis (>3.0%). Patients with glycated hemoglobin (*HbA1c*) ≥ 7% demonstrated a higher proportion of severe fibrinolysis shutdown and a lower proportion of physiologic and partial fibrinolysis compared with those with HbA1c < 7%. Hyperfibrinolysis was infrequent in both groups.

### Multivariable analysis

In the multivariable adjustment, patients with HbA1c ≥ 7% demonstrated a significantly higher predicted probability of severe fibrinolysis shutdown compared with those with HbA1c < 7% (68.8% [95% CI, 51.4-82.2] vs 41.0% [95% CI, 26.3-57.4]; *P* < .001), corresponding to an absolute difference of approximately 28 percentage points (Table III, Fig 2).

**Table III:** Adjusted predicted probability of severe fibrinolysis shutdown by glycemic control

Variable	HbA1c < 7%	HbA1c ≥ 7%	*P* value
Severe fibrinolysis shutdown, adjusted probability, %	41.0 (26.3-57.4)	68.8 (51.4-82.2)	<.001

*HbA1c*, Glycated hemoglobin; *eGFR*, estimated glomerular filtration rate.

Values represent adjusted predicted probabilities with 95% confidence intervals derived from multivariable logistic regression. Model adjusted for age, sex, body mass index, smoking status, renal function (eGFR category), vascular disease burden, antiplatelet regimen, and anticoagulant use. *P* value derived from pairwise comparison of marginal means.

**Fig 2:**
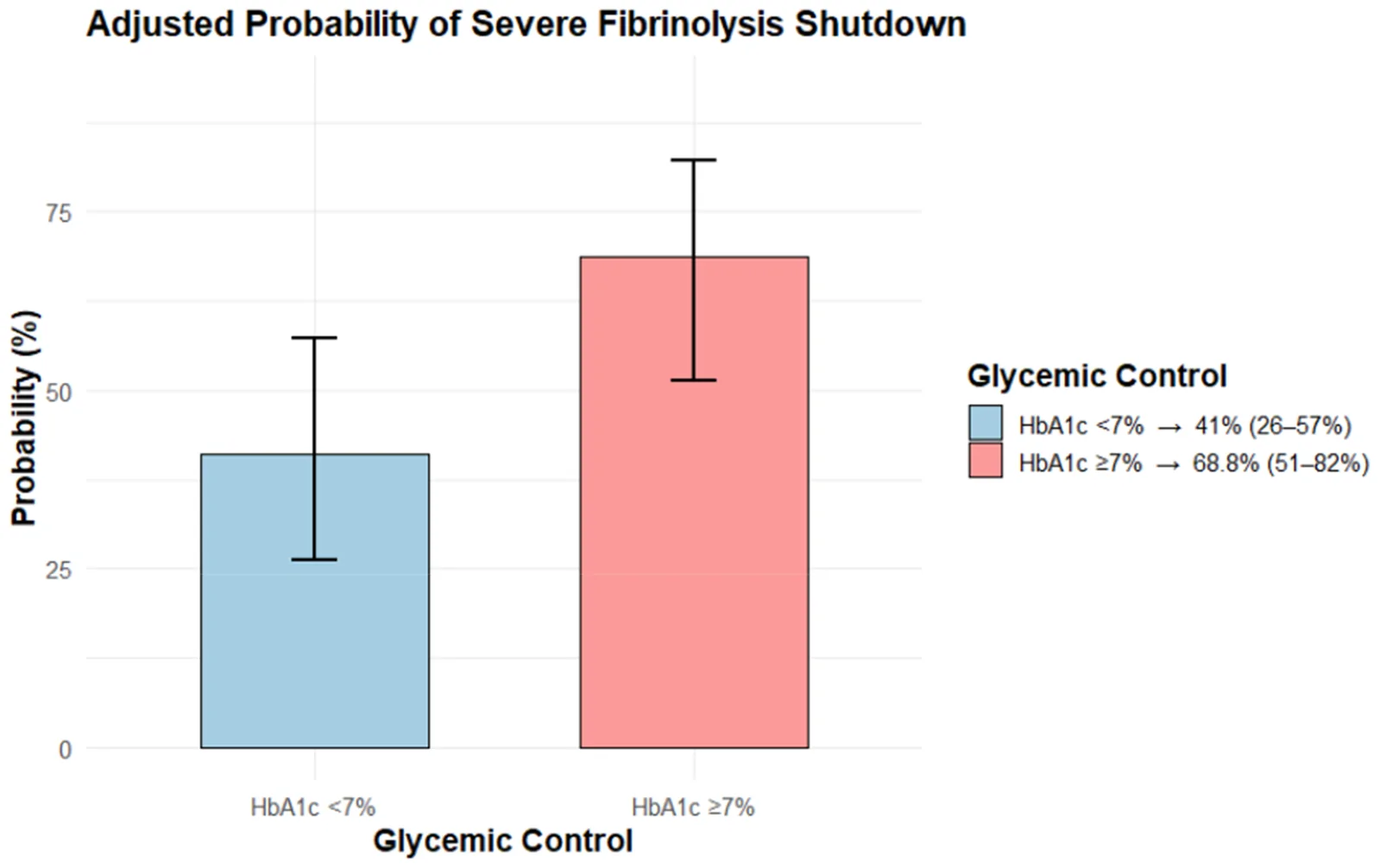
Adjusted probability of severe fibrinolysis shutdown by glycemic control. Bar graph illustrates the adjusted predicted probability of severe fibrinolysis shutdown stratified by glycemic control. Estimates were derived from a multivariable logistic regression model adjusting for age, sex, body mass index, smoking status, renal function, vascular disease burden, antiplatelet therapy, and anticoagulant use. Error bars represent 95% confidence intervals. Patients with glycated hemoglobin (*HbA1c*) ≥ 7% demonstrated a higher probability of severe fibrinolysis shutdown compared with those with HbA1c < 7%.

In continuous analysis, each 1% increase in HbA1c was independently associated with 50% higher odds of severe fibrinolysis shutdown (adjusted OR, 1.50; 95% CI, 1.19-1.93; *P* = .001; Table IV). No other clinical covariates in the multivariable model, including age, sex, BMI, smoking status, renal function, vascular disease burden, antiplatelet therapy, or anticoagulant use achieved independent statistical significance. An inverse relationship between HbA1c and LY30 is illustrated by linear regression in Fig 3.

**Table IV:** Multivariable logistic regression analysis of factors associated with severe fibrinolysis shutdown

Variable	Adjusted OR (95% CI)	*P* value
HbA1c, per 1% increase	1.50 (1.19-1.93)	.001
Age, per year	1.04 (1.00-1.08)	.078
Male sex	0.84 (0.42-1.68)	.621
BMI, per kg/m^2^	1.02 (0.96-1.08)	.593
Smoking status		
Former smoker	0.70 (0.27-1.73)	.452
Nonsmoker	0.61 (0.20-1.78)	.371
Renal function (eGFR, mL/min/1.73 m^2^)		
60-89	0.56 (0.24-1.27)	.164
30-59	0.43 (0.17-1.05)	.066
15-29	0.41 (0.07-2.63)	.325
<15 or dialysis	0.85 (0.25-2.98)	.790
Vascular disease burden		
PAD + CAD/stroke	1.67 (0.76-3.72)	.203
PAD + CAD + stroke	1.26 (0.42-3.87)	.687
Antiplatelet therapy		
Dual antiplatelet	1.50 (0.72-3.20)	.282
None	0.66 (0.24-1.79)	.417
Anticoagulant use	0.67 (0.34-1.30)	.236

*BMI*, Body mass index; *CAD*, coronary artery disease; *CI*, confidence interval; *eGFR*, estimated glomerular filtration rate; *HbA1c*, glycated hemoglobin; *OR*, odds ratio; *PAD*, peripheral artery disease.

HbA1c modeled as a continuous variable; OR represents the change in odds of severe fibrinolysis shutdown per 1% increase in HbA1c. Reference categories: female sex, current smoker, eGFR ≥ 90 mL/min/1.73 m^2^, PAD only, and single antiplatelet therapy.

**Fig 3:**
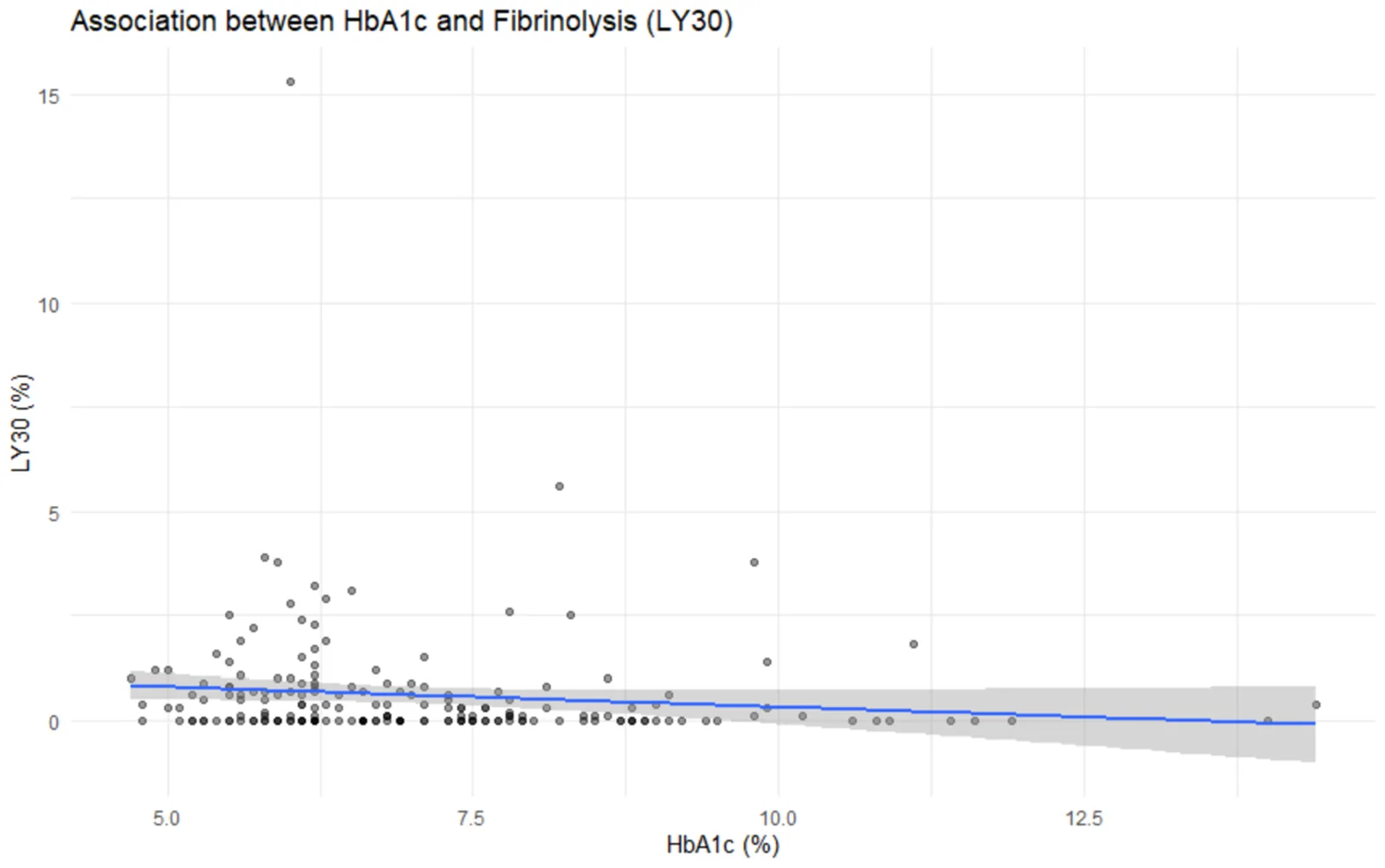
Association between glycated hemoglobin (*HbA1c*) and fibrinolytic activity [lysis at 30 minutes (*LY30*)] assessed by viscoelastic testing. Scatter plot demonstrates the relationship between HbA1c and fibrinolysis, measured by LY30. Each point represents an individual patient. The solid line represents the linear regression fit with corresponding 95% confidence interval (*shaded area*). Higher HbA1c values are associated with lower LY30, indicating impaired fibrinolytic activity and a shift toward fibrinolysis shutdown.

When modeled as a binary exposure, HbA1c ≥ 7% was independently associated with a 3.18-fold increase in the odds of severe fibrinolysis shutdown compared with HbA1c < 7% (adjusted OR, 3.18; 95% CI, 1.64-6.43; *P* < .001; Table V).

**Table V:** Adjusted odds of severe fibrinolysis shutdown by glycemic control (binary analysis)

Variable	Adjusted OR (95% CI)	*P* value
HbA1c ≥ 7% vs <7%	3.18 (1.64-6.43)	<.001

*CI*, Confidence interval; *eGFR*, estimated glomerular filtration rate; *HbA1c*, glycated hemoglobin; *OR*, odds ratio.

HbA1c < 7% was used as the reference category. Model adjusted for age, sex, body mass index, smoking status, renal function (eGFR category), vascular disease burden, antiplatelet therapy, and anticoagulant use.

Glucose-lowering treatment intensity differed significantly by glycemic control. Patients with HbA1c ≥ 7% were more frequently treated with insulin, biguanides, glucagon-like peptide-1 receptor agonists, and dipeptidyl peptidase-4 inhibitors and had a greater overall glucose-lowering medication burden compared with patients with HbA1c < 7% (Table I). In sensitivity analysis additionally accounting for medication burden and insulin therapy, along with the prespecified clinical covariates, HbA1c ≥ 7% remained independently associated with severe fibrinolysis shutdown (adjusted OR, 3.15; 95% CI, 1.40-7.38; *P* = .007). Neither medication burden nor insulin therapy was independently associated with severe shutdown.

### Clinical outcomes associated with severe fibrinolysis shutdown

Clinical outcomes were explored according to severe fibrinolysis shutdown status (Table VI). Thrombosis-driven reintervention occurred significantly more frequently among patients with severe fibrinolysis shutdown than among those without severe shutdown (22.2% vs 8.0%; OR, 3.27; 95% CI, 1.23-10.25; *P* = .010). After adjustment for HbA1c, age, sex, and BMI, severe fibrinolysis shutdown remained independently associated with thrombosis-driven reintervention (adjusted OR, 3.60; 95% CI, 1.42-10.54; *P* = .011). Major amputation (6.0% vs 6.7%; *P* = 1.000) and MACE (13.7% vs 10.7%; *P* = .657) did not differ significantly according to severe shutdown status.

**Table VI:** Association between severe fibrinolysis shutdown and clinically relevant outcomes

Panel A. Clinical outcomes according to severe fibrinolysis shutdown				
Outcomes	No severe shutdown (N = 75)	Severe shutdown (N = 117)	OR (95% CI)	*P* value
Thrombosis-driven reintervention	6 (8.0)	26 (22.2)	3.27 (1.23-10.25)	.010
Major amputation	5 (6.7)	7 (6.0)	0.89 (0.23-3.71)	1.000
MACE	8 (10.7)	16 (13.7)	1.32 (0.50-3.79)	.657

Panel B. Multivariable analysis of thrombosis-driven reintervention		
Variables	Adjusted OR (95% CI)	*P* value
Severe fibrinolysis shutdown	3.60 (1.42-10.54)	.011
HbA1c, per 1% increase	0.91 (0.69-1.17)	.485
Age, per year	0.95 (0.90-0.99)	.015
Sex	0.45 (0.15-1.13)	.107
BMI, per kg/m^2^	1.01 (0.93-1.08)	.865

*BMI*, Body mass index; *CI*, confidence interval; *HbA1c*, glycated hemoglobin; *LY30*, lysis at 30 minutes; *MACE*, major adverse cardiovascular events; *OR*, odds ratio.

Values are presented as n (%) unless otherwise indicated. Severe fibrinolysis shutdown was defined as LY30 ≤ 0.3%. Thrombosis-driven reintervention was defined as repeat surgical or endovascular intervention required for a clinically significant thrombotic event following the index peripheral vascular intervention. Unadjusted comparisons were performed using Fisher exact tests. Multivariable logistic regression was used to evaluate the association between severe fibrinolysis shutdown and thrombosis-driven reintervention after adjustment for HbA1c, age, sex, and BMI.

## Discussion

In this retrospective cohort study of patients with PAD, poor glycemic control was strongly and independently associated with impaired fibrinolysis as measured by viscoelastic testing. Patients with HbA1c ≥ 7% exhibited significantly lower LY30 values, a higher prevalence of severe fibrinolysis shutdown, and a nearly threefold increase in the adjusted odds of this prothrombotic phenotype. Importantly, a continuous, dose-dependent relationship was observed between HbA1c and the odds of fibrinolysis shutdown, suggesting that even incremental elevations in glycemic burden carry clinically meaningful hemostatic consequences. To our knowledge, this represents the first study to characterize the association between glycemic control and fibrinolysis phenotype using viscoelastic testing in a PAD-specific population.

The clinical relevance of fibrinolysis shutdown in PAD likely extends beyond a laboratory abnormality and may represent an important mechanism underlying residual thrombotic risk. Fibrinolysis serves as a critical counterregulatory pathway that limits thrombus propagation and facilitates restoration of vessel patency following vascular injury. Impaired fibrinolysis results in prolonged persistence of fibrin-rich thrombi, creating a prothrombotic environment that may predispose patients to arterial thrombosis and recurrent vascular occlusion. In the setting of PAD and vascular intervention, reduced fibrinolytic capacity may contribute to thrombotic complications, including target-vessel thrombosis, restenosis, graft or stent failure, and ultimately adverse limb events such as recurrent ischemia and major amputation. Although the present study was not designed to evaluate clinical outcomes, the high prevalence of fibrinolysis shutdown observed in this cohort, particularly among patients with poor glycemic control, suggests that impaired fibrinolysis may represent an important biological pathway linking metabolic dysfunction to adverse vascular outcomes and warrants prospective investigation.

The observed association is biologically plausible and mechanistically supported by the existing literature. Hyperglycemia is associated with a hypofibrinolytic state through multiple converging pathways. Foremost among these is the upregulation of PAI-1, the principal inhibitor of tPA and urokinase plasminogen activator. Elevated PAI-1 levels have been consistently demonstrated in states of insulin resistance, type 2 diabetes, and hyperglycemia, and are thought to represent a key mediator of the hypofibrinolytic, prothrombotic phenotype in metabolically dysregulated patients.^8^^,^^9^ In parallel, hyperglycemia leads to the formation of denser fibrin networks through advanced glycation end-products and increased fibrinogen glycation, resulting in clot architectures that are inherently more resistant to plasmin-mediated degradation.^10^^,^^11^ Diminished tPA activity and enhanced platelet activation further compound this prothrombotic milieu.^13^ In this context, an important distinction is the potential influence of chronic glycemic burden compared with acute glucose concentration on fibrinolytic function. HbA1c was selected as the primary glycemic exposure in the present study because it reflects cumulative glycemic control over the preceding approximately 2 to 3 months and is less influenced by transient fluctuations in glucose related to fasting status, meals, physiologic stress, or timing of blood sampling. The consistency of our findings across both categorical and continuous analyses supports an association between chronic glycemic burden and the observed fibrinolytic phenotype. Patients with HbA1c ≥ 7% demonstrated a significantly greater prevalence of severe fibrinolysis shutdown, whereas each incremental increase in HbA1c was associated with progressively greater odds of severe shutdown. This graded relationship suggests that the association extends across the spectrum of chronic glycemic control rather than being limited to a single HbA1c threshold. Contemporaneous glucose measurements were not uniformly available at the time of TEG testing; therefore, prospective studies incorporating simultaneous glucose, HbA1c, and TEG measurements will be important to distinguish the relative contributions of acute and chronic glycemia to fibrinolytic function. Whether the hypofibrinolytic phenotype associated with poor glycemic control is reversible remains unknown. The cross-sectional nature of the present analysis precludes assessment of within-patient changes in fibrinolysis following improvement in HbA1c. Longitudinal assessment of paired HbA1c and TEG measurements before and after improved glycemic control may determine whether fibrinolysis shutdown represents a modifiable phenotype and whether its improvement parallels reductions in chronic glycemic burden. The LY30 parameter measured by TEG integrates these diverse mechanisms into a single, clinically actionable metric of net fibrinolytic capacity, lending face validity to its use as a functional readout of hyperglycemia-mediated hemostatic impairment.

Our findings extend previous work on fibrinolysis shutdown, a phenomenon initially described in the context of trauma and hemorrhagic shock into the domain of chronic arterial disease. In the trauma literature, fibrinolysis shutdown has been associated with increased rates of venous thromboembolism, multiple organ failure, and mortality.^6^^,^^7^ More recently, impaired fibrinolysis has been implicated in the pathogenesis of thrombotic complications in diverse nontrauma settings, including cardiac surgery, obstetric emergencies, and critical illness.^14^^,^^15^ The present study contributes to an emerging body of evidence suggesting that fibrinolysis shutdown may be a generalizable marker of prothrombotic risk that transcends disease context, and that metabolic dysregulation specifically hyperglycemia may be a primary driver in patients with vascular disease.

The dose-dependent nature of the association is particularly noteworthy. The finding that each 1% increment in HbA1c confers a 50% increase in the odds of severe fibrinolysis shutdown suggests a continuous, biologically graded relationship rather than a threshold effect. This has potential implications for how glycemic targets are framed in vascular patients not merely to prevent microvascular or macrovascular complications, but also to minimize the thrombotic consequences of a hypofibrinolytic phenotype. Whether aggressive glycemic optimization can restore fibrinolytic capacity in this population remains an important unanswered question and a potential target for future interventional study.

The predominance of severe fibrinolysis shutdown in the overall PAD cohort present in 60.9% of all patients is itself a striking finding. Even among patients with controlled HbA1c, the majority exhibited a shutdown phenotype, suggesting that PAD itself may be a prothrombotic substrate independent of glycemic status. This is consistent with previous observations of heightened platelet reactivity, increased fibrinogen levels, and impaired clot dissolution in patients with chronic limb ischemia.^3^ The additional burden imposed by hyperglycemia, evident in the substantially higher shutdown prevalence among patients with HbA1c ≥ 7%, may represent a compounding mechanism of thrombotic risk that is not captured by standard clinical assessments or adequately addressed by currently available antithrombotic regimens. Diabetic treatment modality may also contribute to this prothrombotic phenotype.^16^ In a related cohort of diabetic patients with PAD, our group has separately shown that insulin therapy is independently associated with increased arachidonic acid-mediated maximum amplitude clot strength on platelet mapping despite routine aspirin use, suggesting that both impaired fibrinolysis and residual aspirin-sensitive platelet activity may coexist in this population.^16^ Other medication exposures may similarly influence hemostatic profiles in patients with PAD, with serotonergic antidepressant initiation recently associated with greater variability in platelet inhibition following lower-extremity revascularization.^17^

In the present cohort, patients with HbA1c ≥ 7% received more intensive glucose-lowering therapy, including greater use of insulin and multiple glucose-lowering agents. Importantly, however, HbA1c ≥ 7% remained independently associated with severe fibrinolysis shutdown after additional adjustment for medication burden and insulin use (adjusted OR, 3.15; 95% CI, 1.40-7.38). Neither medication burden nor insulin use was independently associated with severe shutdown, suggesting that differences in diabetes treatment intensity do not fully account for the association between chronic glycemic burden and impaired fibrinolysis.

Importantly, the present exploratory clinical outcome analysis suggests that severe fibrinolysis shutdown may have relevance beyond an isolated laboratory phenotype. Patients with severe shutdown experienced a significantly higher incidence of thrombosis-driven reintervention than those without severe shutdown (22.2% vs 8.0%), and severe shutdown remained associated with more than threefold greater odds of this outcome after adjustment for HbA1c, age, sex, and BMI. This finding is biologically consistent with a hypofibrinolytic state, in which impaired clot breakdown may favor persistence of thrombus and contribute to clinically consequential thrombotic failure following peripheral vascular intervention. Although causality cannot be established from the present analysis, the association with thrombotic events requiring repeat intervention provides preliminary clinical support for the relevance of severe fibrinolysis shutdown in PAD.

From a clinical standpoint, these findings raise several important questions. Standard antithrombotic strategies in PAD including single or dual antiplatelet therapy and, in select populations, low-dose anticoagulation principally target platelet activation and thrombin generation, while leaving fibrinolytic pathways largely unaddressed. If fibrinolysis shutdown contributes meaningfully to residual thrombotic risk in this population, therapeutic strategies targeting the fibrinolytic axis such as PAI-1 inhibition or other approaches to restore fibrinolytic capacity warrant investigation. In addition, viscoelastic profiling at the time of clinical evaluation may offer an opportunity to identify patients at heightened thrombotic risk who might benefit from intensified therapy, closer surveillance, or metabolic optimization before vascular intervention.

### Limitations

The retrospective, single-center design precludes causal inference, and limits generalizability. HbA1c reflects mean glycemic exposure over 2 to 3 months and does not capture glycemic variability or acute fluctuations that may independently influence hemostasis. We did not directly measure fibrinolytic mediators such as PAI-1 or tPA, so the proposed mechanistic link remains inferential. Clinical outcome analyses were exploratory and limited by the relatively small number of events. Although severe fibrinolysis shutdown was independently associated with thrombosis-driven reintervention, these findings require validation in larger prospective cohorts with standardized longitudinal follow-up.

## Conclusions

In this cohort of patients with PAD, poor glycemic control was independently and dose-dependently associated with severe fibrinolysis shutdown on viscoelastic testing. Severe fibrinolysis shutdown was additionally associated with a higher incidence of thrombosis-driven reintervention, suggesting potential clinical relevance beyond an isolated laboratory phenotype. These findings identify an association between chronic glycemic burden and impaired fibrinolysis in PAD and support further investigation of fibrinolytic phenotype as a potential marker of thrombotic risk. Prospective longitudinal studies are needed to determine whether improved glycemic control modifies fibrinolytic capacity and whether viscoelastic phenotyping can inform individualized antithrombotic strategies.

## Author Contributions

Conception and design: SB, AD

Analysis and interpretation: SB, AS

Data collection: SB, AS, SF, RB, PL, SP, SV

Writing the article: SB, AS

Critical revision of the article: SB, SF, RB, PL, SP, SV, AD

Final approval of the article: SB, AS, SF, RB, PL, SP, SV, AD

Statistical analysis: SB, AS, SF, RB, PL, SP, SV, AD

Obtained funding: AD

Overall responsibility: AD

## Declaration of generative AI and AI-assisted technologies in the writing process

The authors did not use generative artificial intelligence (AI) or AI-assisted technologies in the writing, analysis, or preparation of this study.

## Data availability

The data that support the findings of this study are available from the corresponding author upon reasonable request.

## Funding

This study was supported by the 10.13039/100000002National Institutes of Health Grant R21 AG077310. The funding source had no role in study design, data collection, data analysis, data interpretation, manuscript writing, or the decision to submit for publication.

## Disclosures

The authors report no conflicts of interest relevant to this study.
